# Effects of a Personalized Stress Management Intervention on Maternal Mental Health: A Randomized Clinical Trial

**DOI:** 10.1007/s00737-025-01619-5

**Published:** 2025-09-17

**Authors:** S. Darius Tandon, Judith T. Moskowitz, Renee C. Edwards, Yudong Zhang, Gina Giase, Brianna Sinche, Abigail L. Blum, S Krislov, Haley M. Reynolds, Aditi Rangarajan, Peter Cummings, Amélie Petitclerc, Nabil Alshurafa, William A. Grobman, Erin A. Ward, Lauren S. Wakschlag

**Affiliations:** 1 Northwestern University Feinberg School of Medicine and Institute for Innovations in Developmental Sciences, Chicago, IL USA; 2 Northwestern University Feinberg School of Medicine, Chicago, IL USA; 3 Northwestern University Feinberg School of Medicine (now at Vanderbilt University), Chicago, IL USA; 4 Northwestern University Feinberg School of Medicine (now at Université Laval), Chicago, IL USA; 5 Northwestern University Feinberg School of Medicine (now at The Warren Alpert School of Medicine, Brown University), Chicago, IL USA; 6 Northwestern University Feinberg School of Medicine (now at Youth Guidance), Chicago, IL USA; 7 Northwestern University Feinberg School of Medicine (now at University of Illinois College of Medicine), Chicago, USA

**Keywords:** Postpartum depression, Just-in-time intervention, Stress

## Abstract

**Purpose:**

While interventions to mitigate and prevent perinatal maternal distress exist, none are personalized based on participants’ daily experiences and intervention response. This study compared maternal distress outcomes (depressive symptoms, anxiety symptoms, perceived stress) between perinatal individuals receiving a personalized mobile health-enhanced cognitive-behavioral intervention and individuals receiving usual prenatal care.

**Methods:**

Pregnant individuals *≤* 22 weeks’ gestation recruited from six prenatal care clinics were randomized to the intervention or usual prenatal care. Intervention participants received a 12-session adaptation of the Mothers and Babies intervention (MB-P), personalized by just-in-time stress reduction and mindfulness content based on elevated physiologic or self-reported stress. Primary outcomes were depressive and anxiety symptoms, and perceived stress. Secondary outcomes were behavioral activation, decentering of thoughts, social support, and mood regulation. Outcomes were measured at baseline, one-week post-intervention, one month postpartum, and three months postpartum. An intent-to-treat approach using mixed-effects models guided analysis.

**Results:**

Forty-nine individuals were randomized to MB-P and fifty-one to usual prenatal care. Participants were 70% White, 33.7 years old on average, and 16.2 weeks gestation. At three months postpartum, intervention participants had lower depressive symptomatology (d = 0.43) and less perceived stress (d = 0.46) than controls. Intervention participants exhibited greater behavioral activation three months postpartum (d = 0.41), greater decentering post-intervention (d = 0.37), and greater mood regulation post-intervention (d = 0.56) and three months postpartum (d = 0.55).

**Conclusion:**

MB-P improved maternal depression and anxiety and mechanisms of behavioral activation, decentering, and mood regulation when compared to usual prenatal care. Future research should examine MB-P impact compared to standard MB without just-in-time content.

Trial registration: Clinical Trials.gov, NCT05052281.

**Supplementary Information:**

The online version contains supplementary material available at 10.1007/s00737-025-01619-5.

## Introduction

Maternal distress encompasses depression, anxiety, and stress in the perinatal period, with each outcome consistently shown to be prevalent and pernicious risk factors for poor maternal wellbeing and child health and development (Brown et al. 2021). Indeed, depression and anxiety are the most common complications of the perinatal period, with the prevalence of depression ranging from 10 to 20%, while approximately 10–13% of perinatal individuals experience maternal anxiety (Viswasam et al. 2019).Maternal distress presents serious health consequences for pregnant individuals, including diminished functioning, impaired interpersonal relationships, less responsive parenting, and—in extreme cases—suicide (O’Hara and McCabe 2013; Tabb et al. 2023). Because maternal distress is also related to impairments in neurodevelopmental outcomes, including decrements in self-regulation and early emerging psychopathology (Clark et al. 2016; Glover 2014; Goodman et al. 2011; Massey et al. 2017; Stein et al. 2014), ameliorating maternal distress has the potential to improve outcomes both perinatal individuals and their children.

Systematic reviews, including a 2019 United States Preventive Services Taskforce (USPSTF) review, highlight effective psychological interventions for preventing perinatal depression (O’Connor et al. 2019). The USPSTF reviewed 50 clinical trials, inclusive of counseling, health system, physical activity, and diet interventions. Counseling interventions were the most common and most effective, demonstrating reduction in the likelihood of the onset of depression by 39%. Based on this evidence, the USPSTF recommended perinatal individuals receive counseling interventions and named Mothers and Babies (MB), specifically, as one of the two most efficacious counseling intervention for preventing perinatal depression. MB is a scalable, manualized intervention based on principles of cognitive-behavioral therapy (CBT) and attachment theory that encourages engagement in pleasant activities, healthy ways of thinking, social support, and awareness of one’s mood. MB has been shown to reduce depressive and anxiety symptoms and prevent new cases of perinatal depression (Le et al. 2011; McFarlane et al. 2017; Muñoz et al. 2007; Tandon et al. 2014, 2021, 2022).

Precision prevention consists of methods that ensure the right intervention is provided to the right population at the right time (Khoury et al. 2016). However, precision approaches have not yet been applied to perinatal depression prevention. Just-in-time adaptive behavioral interventions (JITAIs) allow for personalizing amount and types of intervention content to address variability in participant response to behavioral interventions. JITAIs enhance outcomes since they address the changing needs of individuals in their daily lives across the lifespan of an intervention (Wang and Miller 2020). Recent developments in mobile health (mHealth) technology allow for greater options in delivering personalized JITAI content, which reduces participant burden by integrating intervention practices into their daily lives, thereby expanding access to intervention content beyond in-person delivery (Nahum-Shani et al. 2015). In the United States 97% of women of childbearing age reporting smartphone ownership (Pew Research Center 2024). Perinatal individuals regularly use digital tools to seek pregnancy-related information and are interested in using mobile technologies to address perinatal mental health (Osma et al. 2016). For this study, we generated an innovative, personalized adaptation of MB, using biosensing to detect individual variations in stress physiology along with maternal self-reports of stress, to guide delivery of just-in-time prompts. Biosensing technology is increasingly being used to provide real-time physiological information using dynamic non-invasive measurements of markers including but not limited to heart rate (Kim et al. 2019). This study capitalizes on mHealth technology by using SMS text messages to provide just-in-time content to intervention recipients (see Wakschlag et al. 2021). This study, referred to as Mothers and Babies Personalized (MB-P), is the first to tailor a perinatal mental health intervention (MB) using a JITAI design. Minimizing prenatal stress exposure may impact not only maternal depression and anxiety, but also infant neurodevelopment given the significant influence of the prenatal environment. JITAI designs allow for tailoring MB to respond to individual differences in maternal behavioral and physiologic responses to stress during the perinatal period. In this study, self-reported stress measured via ecological momentary assessment (EMA) and physiological stress measured via biosensing, are used to generate just-in-time content for intervention recipients.

The primary aim of this manuscript is to test the hypothesis that individuals receiving MB-P during pregnancy will report lower levels of depressive symptoms, anxiety symptoms, and perceived stress—each of the three maternal distress distinct constructs—through three months postpartum than individuals receiving usual prenatal care. In doing so, this study fills a gap in the existing perinatal depression intervention literature, as we are unaware of other attempts to use an adaptive trial design to personalize delivery of intervention content. We also examine intervention effects on the cognitive and behavioral mechanisms that are the focus of specific MB-P intervention content, specifically behavioral activation, decentering, social support, and mood regulation. By minimizing maternal distress in the perinatal period using MB-P, this study’s long-term objective is to generate evidence about the potential effects of a personalized intervention on maternal and child health outcomes that can guide researchers and practitioners seeking impactful approaches to mitigating the harmful effects of maternal distress.

## Materials and methods

### Trial design

In this randomized controlled trial (RCT), pregnant individuals were assigned using REDCap’s randomization function via a 1:1 allocation ratio to MB-P or usual prenatal care. Given the primary focus on stress reduction, we stratified (and oversampled) on stress such that half of individuals in each group had elevated baseline stress, defined by a PSS-10 score *≥* 16. Randomization was conducted using the randomization feature in Research Electronic Data Capture (REDCap) (Harris et al. 2019). CONSORT (Moher et al. 2010; Schulz et al. 2010) reporting guidelines for clinical trials were followed.

### Study participants

Pregnant individuals were recruited from six university-affiliated prenatal care clinics in Chicago, Illinois. Participants were eligible if they were *≥* 18 years old, < 22 weeks’ gestation, English-speaking, planning to deliver at the university-affiliated hospital, had access to a smartphone and internet, and agreed to wear a biosensor to track physiologic stress. Individuals were ineligible if they had a significant mental illness or a medical condition documented in their electronic health records that put their child at risk for a neurological disorder. Potential participants were initially recruited using clinic-based recruiters who approached pregnant individuals waiting for their prenatal care appointment and print advertising. When in-person recruitment was suspended due to COVID-19, clinic recruiters identified potential participants and shared information with the study team via secure institutional email. Advertising was also posted on Facebook and Twitter social media platforms. Interested individuals were emailed a REDCap link to complete an online screener that confirmed eligibility, followed by the consent form. Participants were informed during the consent process about procedures to ensure data security and confidentiality, including all information shared during intervention sessions.

### Data collection

#### Primary and secondary outcomes

All study activities were approved by [deleted for blind review] Institutional Review Board (2019–2639). Participant data on primary and secondary outcomes were collected using self-report surveys at baseline (pre-intervention), 1-week post-intervention, and 1-, 3-, 9-, 12-, and 24-months postpartum. This manuscript focuses on maternal outcomes through 3-months postpartum. All data were collected via REDCap or phone (using blinded assessors) in English. Specific instruments to assess primary and secondary outcomes had all been previously used with perinatal populations and were chosen because of their strong reliability and validity in prior studies. Secondary outcomes of behavioral activation, decentering of thoughts, social support, and mood regulation are linked with the specific theoretical mechanisms MB-P is hypothesized to influence.

##### Patient-Reported Outcomes Measurement Information System (PROMIS) depression scale

The 28-item PROMIS depression scale (Choi 2014) assessed presence, frequency, and severity of depressive symptoms. Items were rated from ‘1’ (never) to ‘5’ (always), with higher scores indicating greater symptomatology.

##### State-Trait Anxiety Inventory (STAI) state anxiety scale

The 20-item STAI (Spielberger 1989) assessed severity of state anxiety on a scale from 1 (‘Almost never’) to 4 (‘Almost always’).

##### Perceived Stress Scale 10-item version (PSS-10)

The PSS-10 (Cohen et al. 1983) assessed the degree to which participants appraised situations in their life as stressful, unpredictable, and uncontrollable with higher scores indicating greater perceived stress. Responses fall on a scale from ‘0’ (Never) to ‘4’ (Very Often).

##### Behavioral Activation Depression Scale Short Form (BADS-SF)

The 9-item BADS-SF (Kanter et al. 2009) assessed engagement in positive and enjoyable activities. The BADS consists of nine items rated on a scale from 0 (‘Not at all’) to 6 (‘Completely’), examining changes in behavior activation and avoidance/rumination. Higher total scores indicating greater behavioral activation.

##### Experiences Questionnaire (EQ) 

The EQ (Fresco et al. 2007) is a 20-item instrument that assessed decentering of thoughts and rumination. Decentering is a key construct taught via cognitive restructuring techniques and rumination is a thought pattern that cognitive restructuring is designed to reduce. Items are rated on a scale from ‘1’ (never) to ‘5’ (all the time). Higher scores indicate great ability to decenter one’s thoughts.

##### Medical Outcomes Study Social Support Survey (MOS-SSS) 

The MOS-SSS (Sherbourne and Stewart 1991) consists of 19 items, rated from ‘1’ (none of the time) to ‘5’ (all of the time), measuring the availability of emotional and tangible, with greater scores indicating more perceived social support.

##### Negative Mood Regulation Scale (NMRS)

The 30-item NMRS (Catanzaro and Greenwood 1994; Catanzaro & Mearns, 1990) asked respondents to indicate what they believed they could do when disappointed or experiencing a negative mood on a scale of ‘1’ (strongly disagree) to ’5’ (strongly agree), with higher scores indicating greater ability to regulate one’s mood. *Physiological stress and perceived stress.*

To collect physiological stress data, control and intervention participants wore a BioStamp NPoint sensor (Liu et al. 2018), a flexible patch that attaches to the chest to capture heartrate and heartrate variability. The sensor is worn during the entire waking day, inclusive of showering and exercise. Participants wore the biosensor for 12–15 h a day on a cycle of two weeks on and one week off throughout a 14-week period (corresponding with the time intervention participants were expected to complete MB). A day was labeled physiologically stressful if the total number of stress minutes estimated from the biosensor was > 50%, based on previous published literature (Wakschlag et al. 2021). When participants were physically active, a noise was generated in the ECG signal, which allowed for filtering out this noise prior to detecting stress levels. Participants in both conditions also completed ecological momentary assessments (EMAs) to measure self-reported perceived stress, by responding to text messages with a link to questions on the 4-item Perceived Stress Scale (Cohen et al. 1983). For the 14-week intervention period, EMA messages were delivered four times per day between participant-specified wake and bedtimes via Twilio—a cloud-based communications platform that automates SMS message delivery. Perceived stress data from a given day was averaged to generate a daily perceived stress score, with a score > 4.7 considered to be stressful among perinatal individuals (King et al. 2019).

### Intervention conditions

Intervention participants received MB, a 12-session manualized intervention for perinatal individuals based on principles of cognitive-behavioral therapy (CBT), attachment theory, and psychoeducation (Le et al. 2015; Tandon et al. 2018). Table 1 summarizes core content across sessions; after two introductory sessions, MB provides CBT content related to behavioral activation, identification and reframing unhelpful thought patterns, and promotion of positive interactions with others. Various mindfulness practices, which replaced more generic relaxation activities found in earlier versions of MB, are integrated throughout the 12 sessions. Sessions were delivered 1-on-1 either in-person, by phone, or via Zoom by a trained facilitator; previous MB trials established validity of each delivery modality. Facilitators received MB training prior to implementation and participated in weekly supervision and fidelity checks. To standardize the intervention period for all participants, the 12 MB sessions were delivered over a maximum of 14 weeks, with the first session delivered as close to the baseline assessment as possible to facilitate as much intervention delivery during pregnancy as possible. Intervention participants also received just-in-time (JIT) text messages reinforcing key MB content, encouraging skill practice, and providing links to additional mindfulness and stress reduction content. Sample JIT messages for each MB session are in Table 1; messages were previously developed (Barrera et al. 2019) and adapted for this study by including a fourth text message linked to each session encouraging the use of a mindfulness strategy. Participants received a JIT message associated with their most recent MB session, when they met criteria for elevated stress, as measured by physiologic stress or self-reported perceived stress throughout the day. JIT messages were sent within 24 h of an elevated stress reading. To reduce burden, no JIT message was sent if one had been sent the previous day.

**Table 1:** Intervention content and sample Just-in-Time content, by session

MB Module	MB Session	Core MB Content in Session	Examples of Just-in-Time Content
Introductory	1	♣ Stressors that affect the mother-baby relationship ♣ How Mothers and Babies can help you ♣ What is mindfulness? ♣ Purpose and overview of MB	Everybody has stress. It affects how you feel and can affect your baby. Do something today to manage stress, like taking a few deep breaths.
	2	♣ Your mood and your personal reality ♣ Mindfulness: breath awareness ♣ Quick Mood Scale	Try this guided deep breathing exercise to develop belly breathing.
Pleasant Activities	3	♣ Connection between mood and pleasant activities ♣ Mindfulness: walking meditation ♣ How does what we do affect how we feel?	What do you enjoy doing by yourself and with others? Take a few minutes to do a pleasant activity.
	4	♣ What do you like to do? ♣ Overcoming obstacles to pleasant activities	Pleasant activities can be low to no cost, brief, and things that are part of our daily routines
	5	♣ What do babies like to do? ♣ How do babies learn? ♣ Pleasant activities and my baby	Remember to keep track of how you are feeling (Quick Mood Scale) and home many pleasant activities you do each day
Thoughts	6	♣ Connection between mood and thoughts ♣ What are thoughts? ♣ Mindfulness: sounds and thoughts ♣ Helpful and unhelpful thoughts	When you notice having a negative thought try to replace it with a helpful thought or positive image
	7	♣ Noticing your thoughts ♣ Unhelpful thoughts patterns and talking back ♣ Ways to change unhelpful thoughts that affect my baby and me	Talking back to harmful thoughts is one way to reduce them. Today, change one of your harmful thoughts into a more helpful thought.
	8	♣ Thoughts about being a mother ♣ Goals for my future and my baby’s future	Try this mindfulness practice focusing on compassion.
Contact with Others	9	♣ Connection between mood and contact with others ♣ Breaking the cycle between negative mood and fewer positive contacts	Contact with others can affect your mood. Try to make contact with a positive person in your life.
	10	♣ The people in my life ♣ People in my life and ways they support me	If you had a difficult day, talk to someone you can count on to support you and your baby.
	11	♣ Communication styles and your mood ♣ Getting your needs met ♣ Interpersonal relationships and role changes/transitions ♣ Role disagreements and disputes	It’s important to get your needs met. Positive, clear, and direct requests are effective ways to communicate. Practice being assertive.
Wrap-Up	12	♣ Encourage continued practice of MB skills ♣ Encourage daily mindfulness practice	Congratulations on finishing the MB course! Here are some more mindfulness practices you can try, now or in the future!

Participants in the control condition received usual prenatal care services; usual prenatal care consisted of regular visits of increasingly frequency during pregnancy in accordance with the American College of Obstetricians and Gynecologists (ACOG) recommended schedule of prenatal visits. Control participants also wore biosensors and responded to EMAs but did not receive the MB intervention or JIT content.

### Analysis

To ensure 80% power to detect statistical significance using a 2-sided 5% significance level and minimum clinically relevant difference of 2.9 points in depressive symptoms between intervention and control groups, an analytic sample of 95 participants was required. To allow for approximately 20% of recruited participants to be lost to follow-up, we aimed to recruit 100 individuals. To calculate statistical power, we used the Hotelling-Lawley test in G*Power which considers the increased number of response variables that a mixed-effect size model requires. Whether using the a priori allocation ratio of 1:1 or the post-hoc allocation ratio of 1.06 we achieved 0.8 power in this analysis. While we stratified for baseline stress, we did not stratify analyses based on this variable. Instead, pre-determined stress stratification gave us confidence that the distribution of stress within and between groups would be robust and reduce bias of baseline stress measurements. Using an intent-to-treat approach, mixed-effects models (Hedeker 2006) were used to account for nesting structure of repeated measurements within individuals. Participants with at least one measurement were included in analyses. Missing data were automatically handled by our mixed-effects models. Primary outcomes (depression, anxiety, and stress) were measured at all four timepoints, while secondary outcomes (behavioral activation, decentering, social support, negative mood regulation) were measured at all timepoints except 1-month postpartum. For each outcome, the following predictors were entered in the model: intervention status, time, and the interaction between intervention and time. Time was a continuous variable and one unit of time represented one month (0 = baseline, 4 = post-intervention, 7 = 1-month postpartum, 9 = 3-month postpartum). This coding schema was chosen because on average, the timing of post-intervention, 1-month and 3-month postpartum was about 4, 7, and 9 months after the baseline timepoint. All analyses were conducted in R 4.1.1 (2020).

## Results

### Participant flow and demographics

**Fig. 1:**
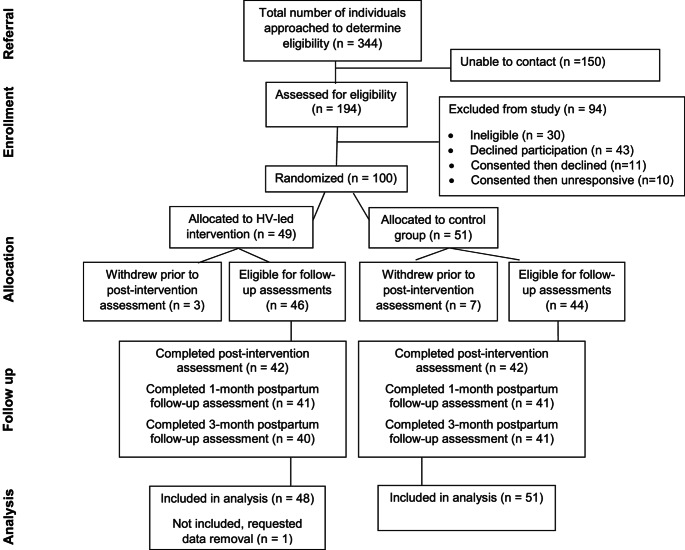
CONSORT flow diagram showing participant flow through each stage of the RCT

Between August 2019 and August 2021, 344 individuals were referred by clinic recruiters and contacted by the research team. Among those assessed for eligibility, 164 met eligibility criteria. Amothose who met eligibility criteria, 121 provided informed consent in REDCap. After consenting, 11 declined participation and 10 could not be reached, yielding 100 pregnant individuals randomized to the intervention (*n* = 49) or control (*n* = 51) group. One intervention participant later withdrew consent and data, yielding a sample of 99 (48 intervention, 51 control) participants; participants lost to follow-up at each time point are depicted in Fig. 1. For each outcome, the percentage of missingness was about 20% at 3-months postpartum (Table S1).

Table 2 depicts the demographic characteristics of the analytic sample. Participants’ mean age was 33.7 years (SD = 4.8) and median weeks’ gestation at baseline was 16.2. Most participants (70%) were White and 13% identified Hispanic/Latina ethnicity. Over 90% had a college degree or more and 84% were married. Study groups were balanced across all demographic characteristics and baseline mental health indicators.

**Table 2:** Participants’ demographics and baseline characteristics

	Control (*n* = 51)	Intervention (*n* = 48)	Overall (*n* = 99)
Demographics	Mean (SD)/No. (%)		
Maternal age at screening	32.95 (4.55)	34.50 (4.96)	33.74 (4.80)
Gestational weeks at enrollment	16.58 (3.66)	15.73 (3.27)	16.17 (3.48)
Mother Race			
White/Caucasian	37 (72%)	32 (67%)	69 (70%)
Asian	5 (10%)	5 (10%)	10 (10%)
Black/African American	4 (8%)	8 (17%)	12 (12%)
More than one race ^a^	X	X	X
Native American/Alaskan Native ^a^	X	X	X
Other ^a^	X	X	X
Unknown ^a^	X	X	X
Mother Ethnicity: Non-Hispanic	41 (80%)	45 (94%)	86 (87%)
Mothers Education college, graduate, or professional degree	48 (94%)	46 (96%)	94 (95%)
Married/Engaged	46 (90%)	42 (88%)	88 (89%)
Household Size	2.84 (1.39)	2.83 (1.28)	2.84 (1.33)
Full-time employment	42 (82%)	37 (77%)	79 (80%)
Income-to-need ratio	5.44 (3.41)	5.49 (3.28)	5.46 (3.33)
Primary and Secondary Outcomes	Mean (SD)/[Min, Max]		
Depressive symptoms ^b^ [Min, Max]	45.95 (14.92) [28.0, 92.0]	49.68 (19.18) [28.0, 104.0]	47.75 (17.13) [28.0, 104.0]
Perceived stress ^c^ [Min, Max]	14.63 (6.16) [1.0, 26.0]	14.78 (7.27) [2.0, 32.0]	14.70 (6.68) [1.0, 32.0]
State anxiety ^d^ [Min, Max]	14.56 (7.73) [1.00,32.00]	15.74 (11.31) [0.00, 45.00]	15.13 (9.59) [0.00, 45.00]
Behavioral activation ^e^ [Min, Max]	36.14 (7.45) [21.00, 53.00]	36.72 (9.35) [15.00, 51.00]	36.42 (8.38) [15.00, 53.00]
Decentering ^f^ [Min, Max]	68.33 (7.73) [53.00, 87.00]	67.55 (8.62) [52.00, 91.00]	67.96 (8.13) [52.00, 91.00]
Social support ^g^ [Min, Max]	4.40 (0.51) [3.00, 5.00]	4.34 (0.66) [2.37, 5.00]	4.37 (0.58) [2.37, 5.00]
Mood regulation ^h^ [Min, Max]	110.35 (13.11) [82.00, 143.00]	110.57 (13.85) [74.00, 134.00]	110.46 (13.40) [74.00, 143.00]

^a^ Too few participants to provide numbers without compromising identifiability ^b^ Baseline depression measured via PROMIS Depression sum score–possible range: [28,140], clinical cutoff 59.7. ^c^ Perceived stress via PSS overall sum score–possible range: [0,40] ^d^ State anxiety via STAI sum score–possible range: [0,60], clinical cutoff 40. ^e^ BADS, Behavioral Activation for Depression scale sum score–possible range: [0,54]. ^f^ EQ, De-centering Experiences Questionnaire sum score–possible range: [0,100]. ^g^ MOS, Medical Outcomes Study overall support index mean score–possible range: [0,5]. ^h^ NMRS, Negative Mood Regulation Scale sum score–possible range: [30,150]

Among intervention participants, the mean number of sessions completed was 10.7 (SD = 3.2, range = 0–12), with 39 (81%) completing all 12 sessions and only one individual receiving no sessions. For those receiving all sessions, the average time to complete sessions was 13.9 weeks. Across all intervention sessions, 56.8% were delivered via video (e.g., Zoom), 25.5% via phone, 17.1% in-person, and 0.6% did not have modality specified. All intervention participants had at least one day with physiological and/or EMA stress data available, which were used to determine need for JIT content. Of all intervention participants, 47/48 (98%) had elevated stress at least once during the intervention period, thereby triggering JIT content. Among intervention participants, the range of JIT messages received was 5–49 (Mean = 25.7; Mode = 14). 

### Primary outcomes

**Fig. 2:**
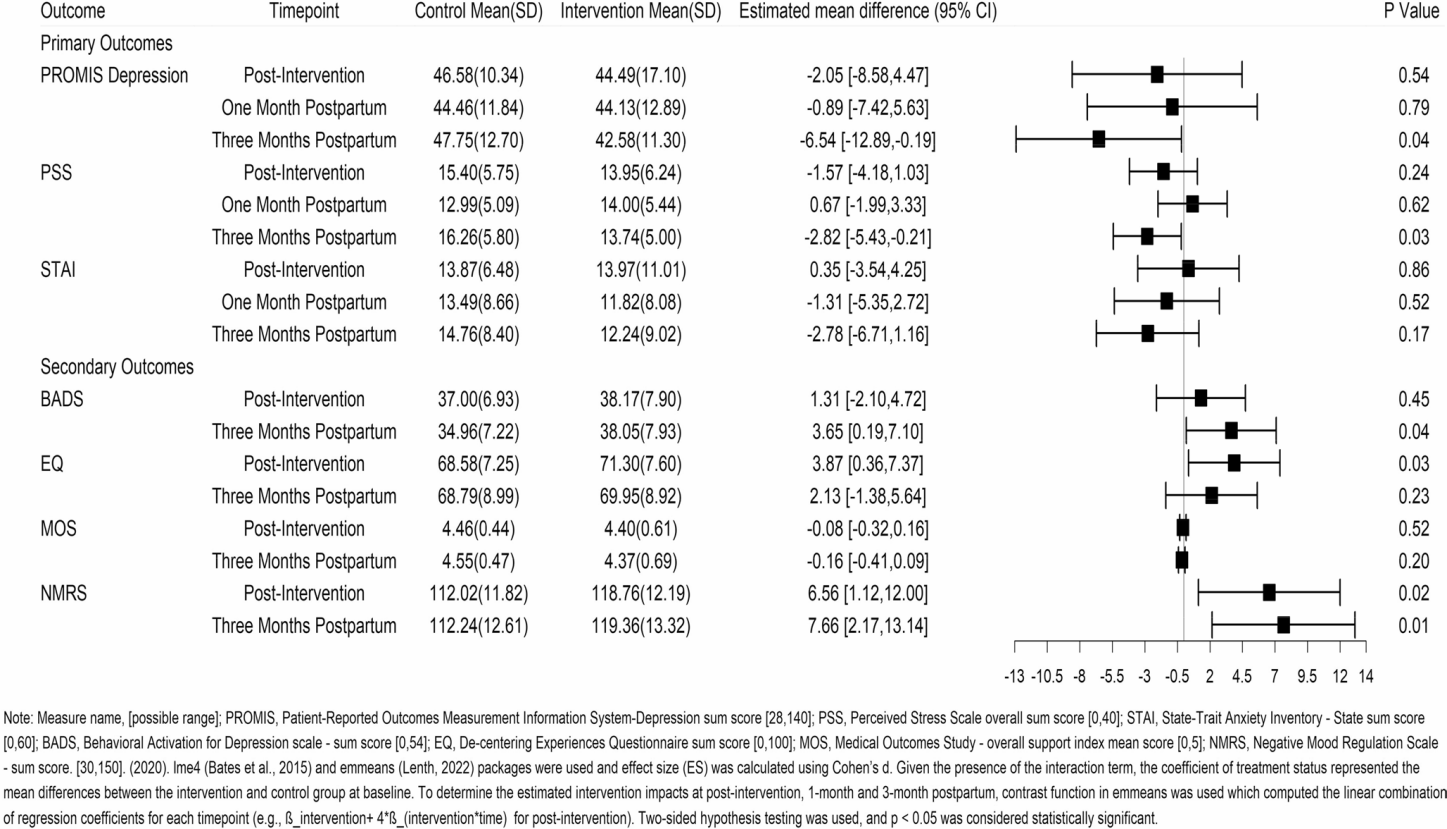
Observed means and estimated mean differences between study groups at post-intervention, 1-month postpartum, and 3-months postpartum

Figure 2 shows the observed means for control and intervention groups, and the estimated mean differences (control vs. intervention) for post-intervention, 1-month, and 3-month postpartum, derived from the random-intercept mixed-effects models (Table S2), for the three primary outcomes. Our central research question was to examine differences between groups at each follow-up time point for primary and secondary outcomes. Intervention impact was demonstrated at 3-months postpartum; compared to the control group, the intervention group had lower depression scores (estimated mean difference ∆, −6.54, 95% CI [−12.89, −0.19], *p* = 0.04, Cohen d = 0.43) and lower stress (∆, −2.82, 95% CI [−5.43, −0.21], *p* = 0.03, Cohen d = 0.46). However, the two groups did not differ on these outcomes at other timepoints. The two groups did not differ in their anxiety symptoms at any timepoint.

#### Secondary outcomes (Mechanisms)

Behavioral activation was significantly higher among intervention participants at 3-months postpartum (∆, 3.65, 95% CI [0.19, 7.10], *p* = 0.04, Cohen d = 0.41). The intervention group also exhibited higher levels of decentering post-intervention (estimated mean difference ∆, 3.87, 95% CI [0.36, 7.37], *p* = 0.03, Cohen d = 0.37). Mood regulation was greater among intervention participants at both post-intervention (∆, 6.56, 95% CI [1.12, 12.00], *p* = 0.02, Cohen d = 0.56) and 3-months postpartum (∆, 7.66, 95% CI [2.17, 12.14], *p* = 0.007, Cohen d = 0.55). At other time points, there were no significant differences in secondary outcomes between the two groups. The two groups did not significantly differ in their social support at any timepoint.

## Discussion and conclusions

This study is the first to our knowledge to conduct a randomized trial of a personalized prenatal stress reduction intervention. Adding to its innovation is the use of an experimental algorithm integrating biosensing and EMA for real time information on maternal experience of distress to guide JITAI content delivery. Given the growing use and of biosensing more broadly in health promotion interventions, this study is also innovative in its efforts to integrate emerging biosensing technologies into perinatal mental health interventions. Recognizing that not all participants benefit equally from perinatal mental health interventions including MB, JITAIs like MB-P allow clinical trials to modify intervention dosage based on participants’ needs, potentially yielding greater benefits compared with more “static” interventions.

Pregnant individuals receiving MB-P reported lower levels of depressive symptoms and perceived stress several months postpartum relative to individuals receiving usual prenatal care. It is important to note that both intervention and usual care participants actively self-monitored stress in response to their EMA and biosensor data, suggesting that intervention effects existed despite the presence of self-monitoring among both study groups. In previous trials, MB delivered without personalized JIT content has been consistently shown to reduce depressive symptoms and perceived stress in perinatal individuals (O’Connor et al. 2019; Tandon et al. 2022). While some studies suggest that self-monitoring of stress can reduce stress and depression by enhancing emotional self-awareness and regulation (e.g. Bakker and Rickard 2018), systematic reviews have not found conclusive evidence in terms of direction or magnitude (Chen et al., 2017; Dogan et al. 2017). In our study, we examined differences between the control and intervention groups in mean levels of stress, depressive symptoms, anxiety, and secondary outcomes at each time point rather than examining within-group changes over time. Although changes from pre- to post-intervention were not explicitly tested, it does not appear that the mean levels of stress and depression in the control group, which only did self-monitoring, decreased.

Intervention participants also exhibited higher levels of decentering, mood regulation, and behavioral activation than individuals receiving usual care. These findings suggest that MB-P was effective in addressing several mechanisms by which improvements in depressive symptoms are thought to occur. These mechanisms are associated with the specific skills that the MB-P intervention discusses, with MB’s theoretical framework suggesting that it is these proximal factors that are associated with maternal distress outcomes of depression, anxiety, and stress. Prior trials have had mixed results examining MB impact on mechanisms of action. It is possible individuals in previous trials not demonstrating improvements in mechanistic outcomes didn’t engage in skill practice between intervention sessions or needed additional reinforcement of key intervention content to effectively put intervention skills into practice. JIT messages in this study provided links to additional MB stress reduction content and reinforced key intervention content, both of which could have increased use of intervention skills translating into improvements in mechanistic outcomes. It is also possible that deploying JIT content within 24 h of elevated stress readings, i.e., when participants were more acutely in need of, and/or likely to be responsive to, JIT content, was instrumental in impacting intervention mechanisms.

Intervention effects on depressive symptoms and perceived stress were detected primarily at 3 months postpartum, consistent with previous MB trials demonstrating effects 3 months or later in the postpartum period (Le et al. 2015; Tandon et al. 2014, 2022). It is possible that intervention effects were not observed at earlier postpartum timepoints due to increases in stress immediately after delivery. Moreover, it is possible that intervention participants likely continue using MB skills over time, leading to greater improvement in mental health outcomes at later data collection time points as participants potentially engage in gradual adoption of intervention skills and begin to see benefits of their cognitive restructuring, behavioral activation, and mood management skills on their depressive symptoms, perceived stress, and everyday interactions with children and adults in their lives. Our observed trend related to intervention impact on anxiety symptoms did not reach statistical significance. Across previous MB trials, one reported effects on anxiety symptoms (Tandon et al. 2018), suggesting that MB may not as effectively address anxiety among perinatal populations. It is also possible that our non-significant results related to anxiety are related to our choice of measurement tools or our sample size.

### Study limitations

Study results should be considered in light of several limitations. Our sample was well-educated and largely non-Hispanic White, which may limit generalizability of our findings to less well-educated and diverse populations. Previous MB trials have been conducted in home visiting, early childhood, and NICU settings which often consist of more economically and racially/ethnically diverse populations, suggesting that future examination of MB-P in such settings could expand generalizability of our findings. It is possible characteristics of our sample facilitated greater engagement with biosensor and EMA data collection, as they may have had fewer life stressors thereby minimizing distractions in biosensor use and EMA response. Our sample limited examination of changes from clinical levels of disorder while enrolled in the trial, as few participants had clinically elevated depression and anxiety at baseline. Additionally, our study design did not allow us to demonstrate incremental utility of a personalized intervention approach. Adaptive trials that can rigorously test the added value of the precision components compared to the standard MB intervention in diverse populations are a key next step.

### Future directions and conclusions

We found that pregnant individuals receiving a personalized postpartum depression intervention experienced lower levels of depressive symptoms and perceived stress relative to individuals receiving usual prenatal care, with additional findings illustrating that several secondary, mechanistic, outcomes influencing these distal outcomes also showed greater improvement among intervention recipients. JITAIs enhances personalization of the MB intervention by delivering real-time support based on each participant’s daily stress levels, as indicated by self-report and physiologic data. This allows intervention content to be delivered at the time an individual is experiencing elevated stress. JITAI messages draw directly from the most recent MB session content, providing skill reinforcement, self-monitoring prompts, mindfulness exercises and coping strategy reminders aligned with the participant’s immediate needs. For example, a participant experiencing high stress after a session focused on social support and contact with others might receive a personalized prompt to reach out to a positive person. This dynamic, timely adaptation to stress is especially well-suited given the fluctuating nature of stress during pregnancy.

Our findings suggest that collection of stress data and deployment of JIT data were feasible and establish the basis for subsequent studies that more fully examine the added value of a personalized intervention approach compared to standard MB intervention. These studies could benefit from enrolling samples with a wider range of baseline depression and anxiety symptoms to generate evidence on MB-P’s efficacy across different severity Levels. Future work should also attempt to examine JIT effectiveness with larger, diverse samples to allow for more robust testing of mediation and to increase generalizability of findings. Our sample continued to complete self-report assessments through 12-months postpartum, It is recommended that other studies similarly follow their samples longitudinally in examining intervention effects, which will allow for more extensive examination of whether improvements in maternal mental health outcomes endured. We are also examining whether improvements in prenatal mental health are related to Less infant dysregulation through 24 months postpartum to understand the potential two-generation effect of intervening prenatally.

## Supplementary Information

Below is the link to the electronic supplementary material.

Supplementary Material 1 (DOCX 32.7 KB)
