# Supplementary material for: Effects of a Personalized Stress Management Intervention on Maternal Mental Health: A Randomized Clinical Trial
Source: Arch Womens Ment Health. 2025 Sep 17;28(6):1585–95. doi: 10.1007/s00737-025-01619-5 (PMC12702803; doi:10.1007/s00737-025-01619-5)
Supplement: Supplementary file 1 — Supplementary Material 1 (DOCX 32.7 KB) [file 737_2025_1619_MOESM1_ESM.docx]

**Table S1**

Percentages of missing data at each wave, split by intervention arm:

|  | Baseline | | Post-Intervention | | 1 Month | | 3 Months | |
| --- | --- | --- | --- | --- | --- | --- | --- | --- |
| Measure | Control (N=51) | Intervention(N=48) | Control (N=51) | Intervention(N=48) | Control (N=51) | Intervention  (N=48) | Control (N=51) | Intervention(N=48) |
|  | N (%) | N (%) | N (%) | N (%) | N (%) | N (%) | N (%) | N (%) |
| PROMIS Depression | 1  (2.0%) | 1  (2.1%) | 15 (29.4%) | 13  (27.1%) | 16 (31.4%) | 12  (25.0%) | 12 (23.5%) | 10  (20.8%) |
| PSS | 0  (0.0%) | 1  (2.1%) | 12 (23.5%) | 10  (20.8%) | 15 (29.4%) | 12  (25.0%) | 12 (23.5%) | 10  (20.8%) |
| STAI | 0  (0.0%) | 1  (2.1%) | 10 (19.6%) | 9  (18.8%) | 16 (31.4%) | 12  (25.0%) | 12 (23.5%) | 10  (20.8%) |
| BADS | 0  (0.0%) | 0  (0.0%) | 10 (19.6%) | 9  (18.8%) | N/A | N/A | 12 (23.5%) | 10  (20.8%) |
| EQ | 0  (0.0%) | 1  (2.1%) | 12 (23.5%) | 10  (20.8%) | N/A | N/A | 12 (23.5%) | 10  (20.8%) |
| MOS | 0  (0.0%) | 0  (0.0%) | 10 (19.6%) | 9  (18.8%) | N/A | N/A | 12 (23.5%) | 11  (22.9%) |
| NMRS | 0  (0.0%) | 0  (0.0%) | 10 (19.6%) | 9  (18.8%) | N/A | N/A | 12 (23.5%) | 10  (20.8%) |

Notes. PROMIS Depression, Patient-Reported Outcomes Measurement Information System-Depression; PSS, Perceived Stress Scale; STAI, State-Trait Anxiety Inventory - State; BADS, Behavioral Activation for Depression; EQ, De-centering Experiences Questionnaire; MOS, Medical Outcomes Study - overall support index; NMRS, Negative Mood Regulation Scale.

Combined:

| Measures | Baseline  (N=99) | Post-Intervention  (N=99) | 1 Month  (N=99) | 3 Months  (N=99) |
| --- | --- | --- | --- | --- |
|  | N (%) | N (%) | N (%) | N (%) |
| PROMIS Depression | 2 (2.0%) | 28 (28.3%) | 28 (28.3%) | 22 (22.2%) |
| PSS | 1 (1.0%) | 22 (22.2%) | 27 (27.3%) | 22 (22.2%) |
| STAI | 1 (1.0%) | 19 (19.2%) | 28 (28.3%) | 22 (22.2%) |
| BADS | 0 (0%) | 19 (19.2%) | N/A | 22 (22.2%) |
| EQ | 1 (1.0%) | 22 (22.2%) | N/A | 22 (22.2%) |
| MOS | 0 (0%) | 19 (19.2%) | N/A | 23 (23.2%) |
| NMRS | 0 (0%) | 19 (19.2%) | N/A | 22 (22.2%) |

Notes. PROMIS Depression, Patient-Reported Outcomes Measurement Information System-Depression; PSS, Perceived Stress Scale; STAI, State-Trait Anxiety Inventory - State; BADS, Behavioral Activation for Depression; EQ, De-centering Experiences Questionnaire; MOS, Medical Outcomes Study - overall support index; NMRS, Negative Mood Regulation Scale.

**Table S2**

Estimated regression coefficients and 95% confidence intervals from random-intercept only mixed-effects models

|  | Treatment  (95% CI) | p-value | Time  (95% CI) | p-value | Treatment x Time  (95% CI) | p-value |
| --- | --- | --- | --- | --- | --- | --- |
| PROMIS Depression | 3.30  (-2.26,8.85) | 0.244 | 0.18  (-0.27,0.64) | 0.430 | -0.96  (-1.61,-0.31) | 0.004 |
| PSS | 0.14  (-2.16, 2.45) | 0.903 | 0.11  (-0.07,0.29) | 0.243 | -0.21  (-0.47,0.05) | 0.106 |
| STAI | 1.53  (-1.94, 5.00) | 0.386 | 0.01  (-0.26,0.27) | 0.970 | -0.44  (-0.81,-0.07) | 0.022 |
| BADS | 0.40  (-2.64, 3.44) | 0.797 | -0.19  (-0.44, 0.05) | 0.121 | 0.34  (-0.01, 0.69) | 0.056 |
| EQ | 0.20  (-2.98, 3.38) | 0.900 | -0.06  (-0.28, 0.17) | 0.611 | 0.31  (-0.01, 0.63) | 0.055 |
| MOS | -0.05  (-0.27, 0.17) | 0.631 | 0.02  (0.00, 0.03) | 0.028 | -0.01  (-0.03, 0.01) | 0.326 |
| NMRS | 1.12  (-3.91, 6.16) | 0.660 | 0.17  (-0.15, 0.49) | 0.304 | 0.82  (0.36, 1.28) | 0.001 |

Notes. PROMIS Depression, Patient-Reported Outcomes Measurement Information System-Depression; PSS, Perceived Stress Scale; STAI, State-Trait Anxiety Inventory - State; BADS, Behavioral Activation for Depression; EQ, De-centering Experiences Questionnaire; MOS, Medical Outcomes Study - overall support index; NMRS, Negative Mood Regulation Scale.

**Table S3**

Estimated regression coefficients and 95% CI from random intercept and slope of time mixed-effects models

|  | Treatment  (95% CI) | p-value | Time  (95% CI) | p-value | Treatment x Time  (95% CI) | p-value |
| --- | --- | --- | --- | --- | --- | --- |
| PROMIS Depression | 3.20 (-3.33 – 9.72) | 0.336 | 0.14 (-0.34 – 0.62) | 0.559 | -0.90 (-1.58 – -0.22) | 0.010 |
| PSS | 0.14 (-2.44 – 2.71) | 0.918 | 0.10 (-0.09 – 0.28) | 0.320 | -0.20 (-0.47 – 0.07) | 0.138 |
| STAI | 1.52 (-2.24 – 5.28) | 0.426 | -0.00 (-0.28 – 0.27) | 0.987 | -0.43 (-0.82 – -0.04) | 0.029 |
| BADS | 0.42 (-2.87 – 3.71) | 0.800 | -0.17 (-0.44 – 0.10) | 0.218 | 0.32 (-0.07 – 0.71) | 0.106 |
| EQ | 0.20 (-2.89 – 3.29) | 0.898 | -0.06 (-0.30 – 0.17) | 0.585 | 0.32 (-0.01 – 0.65) | 0.058 |
| MOS | -0.05 (-0.28 – 0.17) | 0.635 | 0.02 (0.00 – 0.04) | 0.044 | -0.01 (-0.04 – 0.01) | 0.371 |
| NMRS | 1.12 (-3.95 – 6.20) | 0.663 | 0.17 (-0.15 – 0.49) | 0.302 | 0.82 (0.36 – 1.28) | 0.001 |

Notes. PROMIS Depression, Patient-Reported Outcomes Measurement Information System-Depression; PSS, Perceived Stress Scale; STAI, State-Trait Anxiety Inventory - State; BADS, Behavioral Activation for Depression; EQ, De-centering Experiences Questionnaire; MOS, Medical Outcomes Study - overall support index; NMRS, Negative Mood Regulation Scale.
